# Commissioning of the Mobius3D independent dose verification system for TomoTherapy

**DOI:** 10.1002/acm2.12572

**Published:** 2019-03-28

**Authors:** Takumi Kodama, Yoshihiro Saito, Shogo Hatanaka, Masatsugu Hariu, Munefumi Shimbo, Takeo Takahashi

**Affiliations:** ^1^ Department of Radiation Oncology Saitama Cancer Center Saitama Japan; ^2^ Department of Radiation Oncology Saitama Medical Center Saitama Medical University Saitama Japan

**Keywords:** commissioning, independent verification, Mobius3D, tolerance, TomoTherapy

## Abstract

In radiation therapy, a secondary independent dose verification is an important component of a quality control system. Mobius3D calculates three‐dimensional (3D) patient dose using reference beam data and a collapsed cone convolution algorithm and analyzes dose‐volume histogram automatically. There are currently no published data on commissioning and determining tolerance levels of Mobius3D for TomoTherapy. To verify the calculation accuracy and adjust the parameters of this system, we compared the measured dose using an ion chamber and film in a phantom with the dose calculated using Mobius3D for nine helical intensity‐modulated radiation therapy plans, each with three nominal field widths. We also compared 126 treatment plans used in our institution to treat prostate, head‐and‐neck, and esophagus tumors based on dose calculations by treatment planning system for given dose indices and 3D gamma passing rates with those produced by Mobius3D. On the basis of these results, we showed that the action and tolerance levels at the average dose for the planning target volume (PTV) at each treatment site are at *μ* ± 2*σ* and *μ* ± 3*σ*, respectively. After adjusting parameters, the dose difference ratio on average was −0.2 ± 0.6% using ion chamber and gamma passing rate with the criteria of 3% and 3 mm on average was 98.8 ± 1.4% using film. We also established action and tolerance levels for the PTV at the prostate, head‐and‐neck, esophagus, and for the organ at risk at all treatment sites. Mobius3D calculations thus provide an accurate secondary dose verification system that can be commissioned easily and immediately after installation. Before clinical use, the Mobius3D system needs to be commissioned using the treatment plans for patients treated in each institution to determine the calculational accuracy and establish tolerances for each treatment site and dose index.

## 1 INTRODUCTION

In radiation therapy, a secondary independent dose verification of the treatment planning system calculations is an essential part of the quality assurance (QA) process.^1^ The modern treatment planning system (TPS) has many parameters for dose calculations, and it performs complex calculations. Safety and treatment effects may be impaired by incorrect parameters or software malfunctions.

Traditionally, a secondary independent dose verification has been performed using a monitor unit (MU).^1^ This method uses data measured in a water phantom to verify the MU values calculated by the TPS. Before using this system in a clinical environment, it is necessary to spend considerable time and human resources to measure data and verify the accuracy of the dose calculations. Because the TPS and the independent verification system generally require the same measured data, an error in the measurements may propagate into the independent verification system. The verification system thus is not completely independent from the TPS. Moreover, this independent verification system uses a simple dose calculation algorithm, so it can only verify single point doses, and it only works well for homogeneous conditions.^2^

Recently, intensity‐modulated radiation therapy (IMRT) has come into common use for external radiation therapy. TomoTherapy (Accuray Inc., Sunnyvale, CA, USA), which is specifically designed for IMRT, uses a binary Multi Leaf Collimator (MLC) to enable complex beam delivery.^3^, ^4^, ^5^, ^6^ In conditions requiring the complex beam delivery used in IMRT, traditional methods do not have sufficient accuracy for the dose calculations.^7^ Furthermore, MU verification is not adequate to validate TPS dose calculations when the field shapes using the MLC are changed during treatment.

Verification for IMRT is usually performed using measurement‐based techniques.^8^ These methods use a water equivalent homogenous phantom that contains detectors (an ion chamber, film, detector array, etc.) to verify that the dose delivered is the dose planned. For QA, the dose distribution for a patient treatment plan is recalculated from the delivery information obtained from the water‐equivalent phantom. However, the phantom does not represent the actual patient geometry or tissue heterogeneity. These simplifications and the recalculations break the relation between the treatment plan and the QA plan, and any potential error in the patient treatment plan may not propagate to the QA plan. To resolve these issues, techniques using Monte Carlo dose calculation algorithms have been proposed.^9^, ^10^, ^11^ However, it is time consuming to construct the Monte Carlo system and calculate the dose, which limits the applicability of this approach for routine clinical use.

Recently, a new system (Mobius3D; Varian Medical Systems, Inc., Palo Alto, CA, USA) has become commercially available to verify dose calculations. Mobius3D calculates the three‐dimensional (3D) dose distribution for a patient using computed tomography (CT) datasets that employ information from the RT plan after receiving the DICOM CT datasets, RT Plan, RT Structure, and RT dose from the TPS. Following those calculations, Mobius3D automatically compares the dose computed by the TPS with that calculated by Mobius3D. Finally, Mobius3D indicates pass/fail results for the dose‐volume histogram (DVH) limits and the 3D gamma passing rate.^12^, ^13^ The user can check the results in a variety of ways–such as the DVH, dose index, 3D dose distribution, dose profile, and gamma distribution — from any place in the network using a web browser.

Mobius3D has unique characteristics that utilize the reference beam data. The system does not require specific measurement data for TomoTherapy, and it can therefore be installed immediately. Another characteristic is that it uses a collapsed cone convolution superposition algorithm^14^, ^15^ developed by the manufacturer that is accelerated through graphics processing units^16^, ^17^ for the dose calculation. This algorithm can produce accurate calculations for IMRT and for heterogeneous conditions.^18^ These two characteristics make it possible for a user to validate the TPS dose calculation and provide 3D dose distributions and DVH information immediately and independently from the TPS.

The accuracy of the dose calculation from a verification system must be commissioned and validated before clinical use. Commissioning and clinical implementation for Mobius3D has been reported for C‐arm linear‐accelerator photon beams,^19^, ^20^, ^21^ but this has not previously been done for TomoTherapy. Mobius3D includes parameters for modifying the initial registered data if the machine data in the user institution do not correspond to the reference beam data. The purpose of the present work is to describe our experience with these adjustment parameters and to evaluate the accuracy of dose calculations for TomoTherapy. We also indicate the action and tolerance levels for clinical implementation.

## 2 MATERIALS AND METHOD

### 2.A Mobius3D system for TomoTherapy

The TomoTherapy system has three nominal field widths (NFWs) in the superior–inferior direction of the patient: 1.0, 2.5, and 5.0 cm jaw size at the source‐to‐axis distance 85 cm. To adjust the reference beam data, Mobius3D has three parameters for TomoTherapy:

- the calibration value,
- the output factor for NFW 1.0 cm × 40 cm normalized to NFW 5.0 cm × 40 cm, and
- the output factor for NFW 2.5 cm × 40 cm normalized to NFW 5.0 cm × 40 cm.

The output factor for NFW 5.0 cm × 40 cm is locked at 1.0, and the user cannot change this in Mobius3D. If this output factor needs to be changed, the Mobius support team must provide a new license file that has an arbitrary calibration value. This file affects all the NFW conditions, so the user must recalculate and validate all plans. Thus, to adjust the parameters for all NFW conditions, it is first necessary to prepare the license file adjusted to NFW 5.0 cm and then adjust the two output factors to NFW 1.0 and 2.5 cm.

### 2.B Dosimetric verification

We used a water equivalent phantom called “cheese phantom” that was provided by Accuray for our TomoTherapy. This phantom is a cylinder 30 cm in diameter and 18 cm long. It can contain a cylindrical ion chamber that can be positioned at an arbitrary location along the perpendicular axis to the cylinder axis and a film in half of the phantom. To evaluate the dose calculation accuracy for patient plans, we imposed the contours of patients who have been treated at our institution on this phantom. In the present study, we evaluated nine helical IMRT plans for each NFW. These plans include three each for prostate, head‐and‐neck, and pelvic lymph node cases. We calculated these plans using Planning Station version 5.1.0.4 on the basis of our clinical dose constraints, and we sent the DICOM datasets to Mobius3D to calculate the verification dose. We used an ion chamber with an active volume of 0.057 cc (Exradin A1SL ionization chamber; Standard Imaging Inc., Middleton, WI, USA) to measure two points in the PTV for comparison with the doses calculated by Planning Station and by Mobius3D. We contoured the A1SL sensitivity volume in the PTV and recorded the mean dose delivered to that volume. These volumes were placed at a low gradient region such that the ratio of maximum dose to minimum dose in the sphere of radius 7.0 mm centered at the sensitivity volume was over 0.96.^22^ We defined the dose difference ratio between measurement and calculation as follows:

(1) $$\text{Dose difference ratio}=\frac{D_{\mathrm{calc}}-D_{\mathrm{meas}}}{D_{\mathrm{meas}}}\times 100\left[\%\right],$$

where *D*_calc_ is the dose as calculated by Mobius3D or Planning Station, and *D*_meas_ is the dose as measured using the ion chamber. To measure the relative dose distribution, we used GAFCHROMIC EBT3 Film (Ashland ISP Advanced Materials, NJ, USA). A film was placed in the sagittal plane to measure the planar dose distribution. We extracted the calculated dose distribution from the DICOM RT dose using an in‐house program, and we performed gamma analysis using the RIT 113 film dosimetry system (Radiological Imaging Technology, Inc., Denver, Colorado, CO, USA). Each dose distribution was normalized to the mean dose of the area in the PTV. We performed gamma analysis using the criteria of 3% and 3 mm, which are the dose difference relative to the global maximum dose and the distance‐to‐agreement values, respectively, using a 10% threshold to exclude the low‐dose region. We calculated the four indices mean (*μ*), standard deviation (*σ*), maximum (*Max*), and minimum (*Min*) for each dose difference ratio and gamma passing rate.

### 2.C Clinical implementation

To evaluate the differences between Mobius3D and Planning Station in a clinical situation, we recalculated the treatment plans using Mobius3D for patients who had been treated in our institution. This study was approved by the institutional review board in our institution (number 836). We included the following numbers of plans and treatment sites, with the corresponding NFWs: 29 prostate plans with NFW 1.0 cm, 27 prostate plans with NFW 2.5 cm, 62 head‐and‐neck plans with NFW 2.5 cm, and 8 esophagus plans with NFW 2.5 cm. Prescription protocol was that 95% of the PTV was received as the prescription dose. Mobius3D recalculated the 3D dose distribution without renormalization. We obtained the following dose indices for the PTV (*D*_mean_, *D*_2%_, *D*_50%_, *D*_95%_, and *D*_98%_), rectum (*D*_mean_, *V*_65Gy_, and *V*_40Gy_), bladder (*D*_mean_, *V*_65Gy_, and *V*_40Gy_), spinal cord (*D*_max_), each parotid (*D*_mean_), and each lung (*D*_mean_, *V*_20Gy_, and *V*_5Gy_). We defined the dose difference ratios and volume differences for each dose index between Planning Station and Mobius3D as follows:

(2) $$\text{Dose difference ratio}=\frac{D_{M3D}-D_{\mathrm{PS}}}{D_{\mathrm{ref}}}\times 100\left[\%\right],\text{and}$$

(3) $$\text{Volume difference}=V_{M3D}-V_{\mathrm{PS}}\left[\%\right].$$

where *D*_M3D_ is the dose as calculated by Mobius3D, *D*_PS_ is the corresponding dose as calculated by Planning Station, *D*_ref_ is the maximum point dose in the patient as calculated by Planning Station, *V*_M3D_ is the ratio of the volume of the organ at risk (OAR), which received an equal or greater arbitral dose, to the whole volume, as calculated by Mobius3D, and *V*_PS_ is the corresponding volume ratio as calculated by Planning Station. To evaluate the 3D dose distribution, we performed 3D gamma analysis for the criteria of 5%/3 mm and 3%/3 mm, both with a 10% threshold. We calculated the four indices *μ*,* σ*,* Max*, and *Min* for each dose difference ratio and volume difference.

### 2.D Action and tolerance level

The action and tolerance levels for the dose differences between Planning Station and Mobius3D were set at *μ ± 2σ* and *μ ± 3σ*, respectively. We calculated the mean dose for the PTV at each treatment site and the mean dose for the OAR at all treatment sites.

## 3 RESULTS

### 3.A Dosimetric verification

In this report, we optimized three parameters on the basis of verification using the ion chamber for each NFW. We adjusted the calibration value, which we increased to 3% from the initial registered data, and we increased the output factor for NFW 1.0 cm by 2.5% from initial registered data. Table 1 shows the differences between the ion chamber measurements and the calculations by Mobius3D and Planning Station after changing these parameters. We performed this comparison for two points in the PTV, and the mean values are listed. For all cases, the dose difference ratio [*μ ± σ* (*Min* to *Max*)] was −0.2 ± 0.6% (−1.6 to 1.2) for Mobius3D and was −1.0 ± 1.0% (−2.8 to 0.6) for Planning Station. In this result, the Planning Station slightly underestimated the dose compared to measurement using ion chamber. Especially for prostate cases with NFW 5.0 cm, differences between Planning Station and measurement were from −2.5 to −2.7. The standard deviation is similar to Mobius3D. Table 2 shows the gamma passing rates using the criteria of 3%/3 mm between measurements with GAFCHROMIC EBT3 and calculations using Mobius3D and Planning Station. For all cases, the gamma passing rates were 98.8 ± 1.4% (94.6 to 100.0) and 97.9 ± 2.2% (92.5 to 99.8) for Mobius3D and for Planning Station, respectively. In this result, no significant differences were observed for prostate and head‐and‐neck cases at both systems. However, passing rates were slightly lower at Planning Station than Mobius3D for pelvic lymph node cases. The failures of gamma evaluation were observed at high‐dose gradient region beside PTV and at low‐dose region in the organ at risk.

**Table 1:** Summary of differences between measurements using ion chamber and calculations by two systems

	Difference (%)					
	NFW 1.0 cm		NFW 2.5 cm		NFW 5.0 cm	
	M3D^a^	PS^b^	M3D	PS	M3D	PS
Treatment site						
Prostate 1	−0.5	−1.3	−0.7	−1.6	−0.4	−2.7
Prostate 2	−0.3	−1.3	−0.6	−1.7	−0.5	−2.7
Prostate 3	−0.1	−1.3	−0.3	−1.3	−0.1	−2.5
Head & neck 1	−0.5	−0.1	−0.4	−0.3	−1.1	−2.4
Head & neck 2	0.6	−0.1	0.3	−0.8	0.9	−1.7
Head & neck 3	0.7	0.1	0.7	−0.4	0.9	−1.8
Pelvic lymph node 1	0.0	0.3	−0.2	−0.1	−0.1	−1.1
Pelvic lymph node 2	−0.5	0.4	−0.8	−0.2	−0.7	−1.3
Pelvic lymph node 3	−1.0	0.3	−0.8	−0.5	−0.1	−1.3
Statistical indices						
*μ*	−0.2	−0.3	−0.3	−0.8	−0.1	−1.9
*σ*	0.6	0.8	0.6	0.6	0.8	0.6
Max	0.9	0.6	0.8	0.1	1.2	−1.0
Min	−1.6	−1.4	−1.3	−1.8	−1.2	−2.8

a = Mobius3D; b = Planning Station.

**Table 2:** Summary of the gamma passing rates between measurement using film and calculations by two systems

	Gamma passing rates (the criteria of 3%/3 mm) (%)					
	NFW 1.0 cm		NFW 2.5 cm		NFW 5.0 cm	
	M3D^a^	PS^b^	M3D	PS	M3D	PS
Treatment site						
Prostate 1	97.2	99.3	99.0	97.7	99.8	99.6
Prostate 2	99.8	99.7	99.9	99.7	99.8	98.6
Prostate 3	100.0	99.6	100.0	99.5	99.7	99.5
Head and neck 1	98.7	98.4	98.7	98.4	99.6	97.3
Head and neck 2	99.6	99.8	98.8	98.3	97.7	99.3
Head and neck 3	100.0	99.7	99.5	99.0	99.6	98.9
Pelvic lymph node 1	99.7	98.7	99.3	99.0	97.9	93.0
Pelvic lymph node 2	97.0	92.5	94.6	94.4	97.3	93.7
Pelvic lymph node 3	99.5	97.9	95.6	96.1	98.4	96.9
Statistical Indices						
*μ*	99.1	98.4	98.4	98.0	98.8	97.4
*σ*	1.2	2.3	1.9	1.7	1.0	2.5
Max	100.0	99.8	100.0	99.7	99.8	99.6
Min	97.0	92.5	94.6	94.4	97.3	93.0

a = Mobius3D; b = Planning Station.

### 3.B Clinical implementation

**Figure 1:**
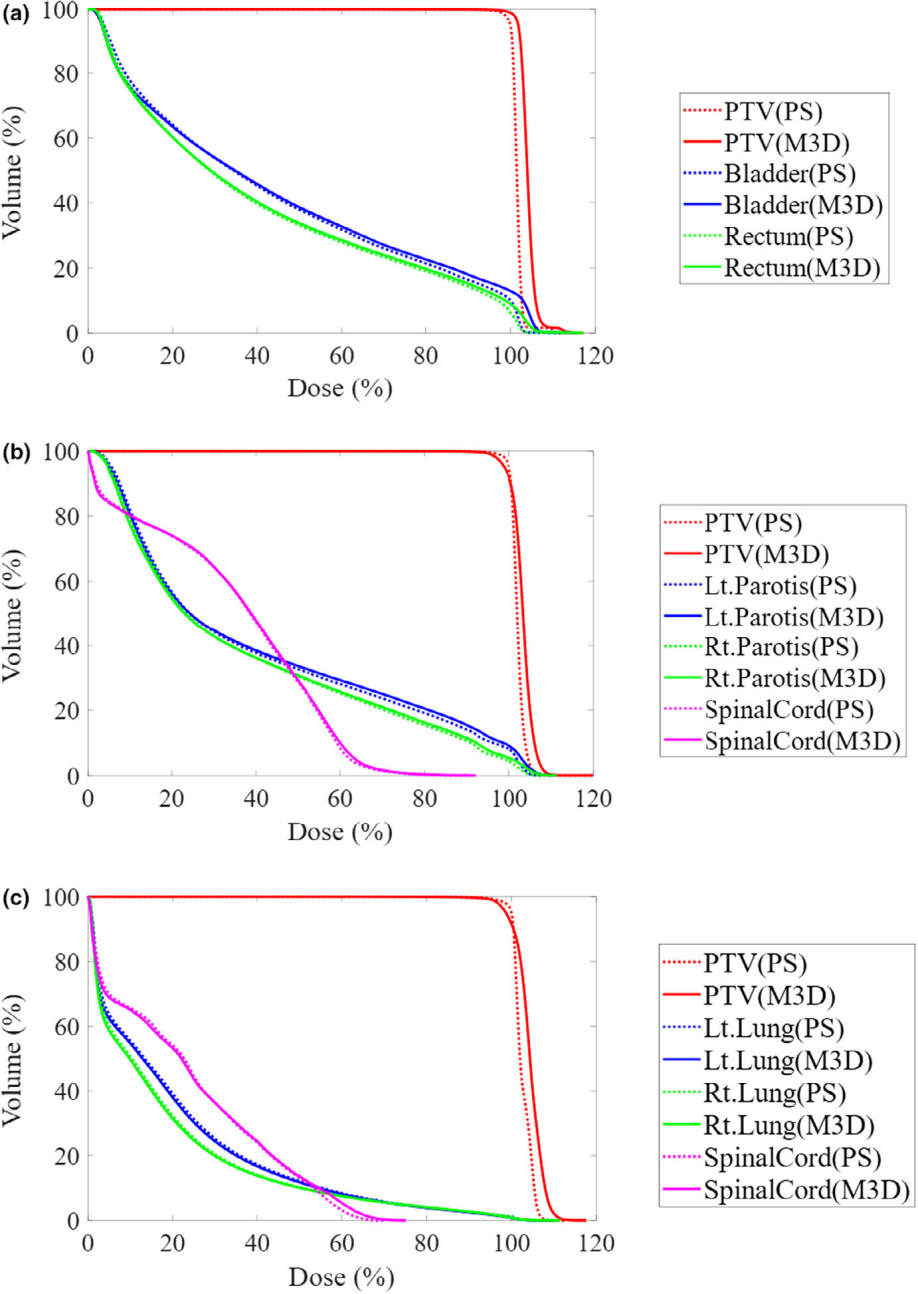
The mean dose‐volume histogram calculated by Mobius3D (M3D) and Planning Station (PS) at (a) the prostate, (b) the head‐and‐neck, and (c) the esophagus.

Figure 1 shows the mean DVH calculated by Mobius3D and by Planning Station for both NFW 1.0 cm and 2.5 cm at the prostate, for NFW 2.5 cm at the head‐and‐neck area, and for NFW 2.5 cm at the esophagus. The Mobius3D values were slightly larger and less homogeneous compared to Planning Station for PTV. Table 3 shows a summary of the differences for each dose index between Mobisu3D and Planning Station. No large difference was observed between NFW 1.0 and 2.5 cm in the prostate plans, but differences were observed at *D*_2%_ for PTV at all treatment sites. At the OAR, differences were within 5% for all DVH indices and were mostly within 3%. In Table 1, calculated dose by Planning Station was higher than measured dose by about 1.0% in the case of prostate with NFW 1.0 and 2.5 cm and of head‐and‐neck with NFW 2.5 cm. This states that commissioning accuracy for each system affects independent dose verification results.

**Table 3:** Summary of the differences in dose indices between Mobius3D and Planning Station

Structures		Difference (%)							
	Dose indices	Prostate				Head & neck			
		NFW 1.0 cm (n = 29)		NFW 2.5 cm (n = 27)		NFW 2.5 cm (n = 62)		NFW 2.5 cm (n = 8)	
		*μ* ± *σ*	(*Max*/*Min*)	*μ* ± *σ*	(*Max*/*Min*)	*μ* ± *σ*	(*Max*/*Min*)	*μ* ± *σ*	(*Max*/*Min*)
PTV	*D*_mean_	2.3 ± 0.4	(3.4/1.4)	2.6 ± 0.4	(3.5/1.6)	1.1 ± 0.6	(2.5/−0.5)	1.6 ± 0.8	(3.2/0.6)
	*D*_2%_	3.7 ± 0.6	(4.9/2.3)	4.2 ± 0.8	(6.3/2.5)	2.7 ± 0.7	(4.5/1.5)	3.6 ± 1.0	(5.5/2.5)
	*D*_50%_	2.1 ± 0.5	(3.3/1.1)	2.5 ± 0.4	(3.3/1.5)	1.2 ± 0.6	(2.5/0.0)	1.8 ± 0.8	(3.6/0.8)
	*D*_95%_	2.0 ± 0.6	(3.3/0.5)	2.2 ± 0.6	(3.2/1.0)	−0.8 ± 1.2	(1.8/−4.3)	−1.0 ± 1.0	(0.2/−2.9)
	*D*_98%_	2.1 ± 0.5	(3.5/1.1)	2.3 ± 0.8	(3.6/−0.2)	−1.2 ± 1.6	(1.8/−6.6)	−1.3 ± 1.6	(1.4/−3.2)
Rectum	*D*_mean_	0.5 ± 0.6	(1.8/−0.2)	0.6 ± 0.9	(2.0/−1.6)	–	–	–	–
	*V*_65Gy_	−0.8 ± 0.9	(0.6/−3.0)	−0.8 ± 1.0	(1.1/−3.1)	–	–	–	–
	*V*_40Gy_	−0.7 ± 0.9	(0.6/−2.5)	−0.7 ± 1.2	(1.9/−3.0)	–	–	–	–
Bladder	*D*_mean_	0.7 ± 0.6	(2.1/−0.2)	0.8 ± 0.4	(1.6/0.0)	–	–	–	–
	*V*_65Gy_	−1.4 ± 0.8	(−0.1/−4.1)	−1.4 ± 0.6	(−0.6/−3.2)	–	–	–	–
	*V*_40Gy_	−0.7 ± 0.5	(−0.1/−1.9)	−0.9 ± 0.4	(−0.2/−1.6)	–	–	–	–
Spinal cord	*D*_max_	–	–	–	–	1.2 ± 0.9	(3.0/−0.5)	1.0 ± 1.3	(3.5/0.0)
Left parotids	*D*_mean_	–	–	–	–	0.7 ± 0.7	(2.2/−0.4)	–	–
Right parotids	*D*_mean_	–	–	–	–	0.2 ± 0.6	(1.8/−0.6)	–	–
Left. lung	*D*_mean_	–	–	–	–	–	–	−0.5 ± 0.2	(−0.3/−0.9)
	*V*_20Gy_	–	–	–	–	–	–	−0.4 ± 0.4	(0.0/−1.0)
	*V*_5Gy_	–	–	–	–	–	–	−1.2 ± 0.5	(−0.6/−2.0)
Right lung	*D*_mean_	–	–	–	–	–	–	−0.4 ± 0.1	(−0.3/−0.7)
	*V*_20Gy_	–	–	–	–	–	–	−0.3 ± 0.2	(−0.1/−0.8)
	*V*_5Gy_	–	–	–	–	–	–	−1.2 ± 0.6	(−0.5/−2.0)

Table 4 shows a summary of the gamma passing rates using the criteria of 5%/5 mm and 3%/3 mm between Mobius3D and Planning Station. No larger difference was observed between NFW 1.0 and 2.5 cm in the prostate plans. Gamma passing rates using the criteria of 3%/3 mm were slightly lower for head‐and‐neck and esophagus cases compared to prostate cases.

**Table 4:** Summary of the three‐dimensional (3D) gamma passing rates between Mobius3D and Planning Station

Treatment site	NFW	3D gamma passing rates (%)			
		5%/3 mm		3%/3 mm	
		*μ* ± *σ*	(*Max*/*Min*)	*μ* ± *σ*	(*Max*/*Min*)
Prostate	1.0 cm	99.8 ± 0.2	(100.0/99.2)	98.6 ± 1.0	(99.5/95.4)
	2.5 cm	99.9 ± 0.1	(100.0/99.6)	98.5 ± 0.6	(99.6/96.5)
Head and neck	2.5 cm	99.3 ± 0.3	(99.9/98.6)	96.8 ± 1.3	(99.0/93.6)
Esophagus	2.5 cm	99.1 ± 0.4	(99.9/98.7)	96.4 ± 1.6	(99.0/94.4)

### 3.C Action and tolerance level

**Figure 2:**
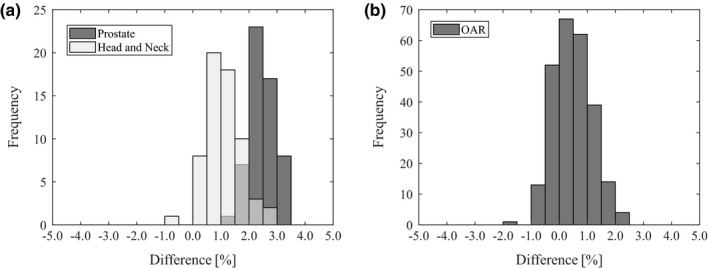
Distribution of the mean dose discrepancies between Mobius3D and Planning Station for (a) the planning target volume at the prostate and the head‐and‐neck and (b) for the organ at risk (OAR) at all treatment sites.

Figure 2 shows a histogram of the mean dose discrepancies for the PTV and the OAR between Mobius3D and Planning Station. The standard deviation was similar, however, the median is different from the head‐and‐neck plans in Fig. 2(a). Table 5 shows the action and tolerance levels, which was set at *μ ± 2σ* and *μ ± 3σ*, respectively, for the mean dose discrepancies between Mobius3D and Planning Station.

**Table 5:** The action and tolerance levels for the mean dose discrepancies between Mobius3D and Planning Station

Treatment site	Action Level *μ* ± 2*σ* (%)	Tolerance level *μ* ± 3*σ* (%)
Prostate	1.6 ~ 3.4	1.1 ~ 3.8
Head and neck	−0.1 ~ 2.3	−0.7 ~ 2.9
Esophagus	0.1 ~ 3.1	−0.7 ~ 3.9
OAR	−0.9 ~ 1.8	−1.6 ~ 2.5

## 4 DISCUSSION

Using reference beam data has the benefits that the verification system is independent from the TPS and that is can be implemented immediately in the clinic. It is important that beam data of linear accelerator in the user institution be consistent with the reference beam data in Mobius3D. In this work, we used the method written in the Mobius User Manual to commission Mobius3D for TomoTherapy. The vender recommends adjusting the parameters if the difference between measurements using an ion chamber and calculations by Mobius3D exceeds 2%. We investigated the parameters based on the results at three treatment sites with three NFWs. Mobius3D has only three parameters, but significant differences were not observed in point dose and planar dose comparisons for measurements and calculations in our phantom study. These results are similar to previous study using ion chamber and water‐equivalent phantom for C‐arm linear accelerator.^20^, ^21^, ^23^, ^24^ Conversely, point dose using Planning Station was underestimated compared to measurement for prostate cases with NFW 5.0 cm as listed in Table 1. In our institution, Planning Station with NFW 5.0 cm was adjusted for not only a short target in the superior–inferior direction of the patient like prostate but also for a long target like craniospinal. Planning Station was optimized for a many types of clinical treatment situations. In Table 2, gamma passing rates were lower compared to Mobius3D for pelvic lymph node cases. To be completely independent from the TPS, implementations in Mobius3D were developed by the manufacturer. The calculation algorithm specification in the Mobius3D manual for MLC leakage is considered to be 0.25% in Mobius3D, whereas this is not considered in Planning Station. In addition, the source size is around 1 mm in Mobius3D, whereas a point source is assumed in Planning Station. These parameters have indicated using Monte Carlo simulation and direct measurement methods in previous reports.^25–28^ Differences in these two implementations were more apparent in pelvic lymph cases. Source size affects penumbra shape and MLC leakage affects organ at risk.^29^, ^30^

**Figure 3:**
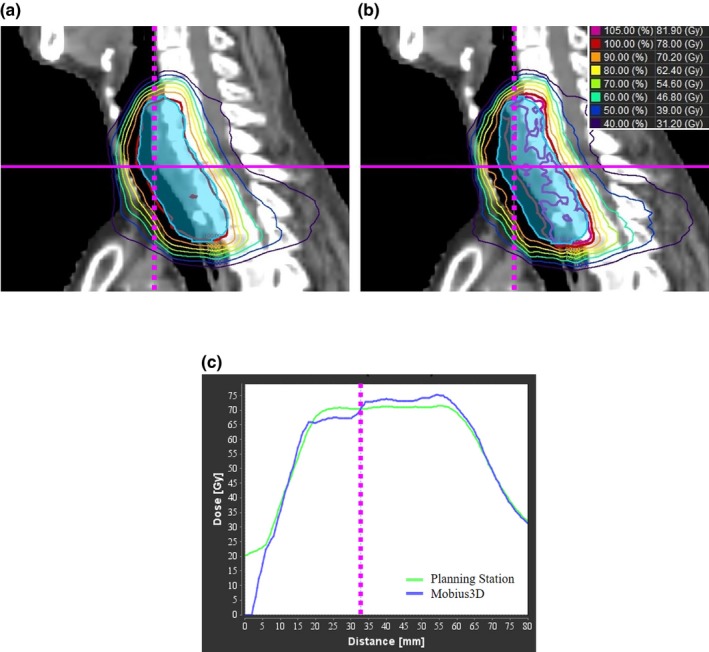
Isodose distributions at esophagus case calculated by (a) Planning Station and (b) Mobius3D, and dose profiles at magenta line (solid). Magenta lines (dashed) in the dose profiles indicate the interpoint section of solid line and dashed line in the isodose distributions.

We have also determined the differences between Mobius3D and Planning Station for the DVH, dose indices, and 3D gamma passing rates obtained from treatment plans for patients treated in our institution. Mobius3D values were slightly larger and less homogeneous compared to Planning Station. They resulted from differences in the commissioning accuracy, beam data (profiles and implemented parameters for MLC), calculation algorithm specifications, and CT number‐to‐physical‐density conversion table (CT‐PD) between Mobius3D and Planning Station. The effect of commissioning accuracy and beam data is indicated in the phantom study. In Table 1, Mobius3D values were larger than Planning Station at point dose in PTV. In addition, calculation accuracy in heterogeneity condition affects these results in the patient study. Figure 3 shows the isodose distributions and dose profiles of the esophagus case calculated by Planning Station and Mobius3D. Blue color wash represents PTV. PTV was covered by prescription dose in Planning Station, however, PTV was not covered at low‐density regions in Mobius3D and there are hot regions at tissues. More details are shown as profiles in Fig. 3(c), Mobius3D calculated lower than Planning Station at left side from dashed line, which is the low‐density region. Conversely, Mobius3D calculated higher than Planning Station at the right side from the dashed line. TomoTherapy has different structures specified for helical IMRT, but similar differences with heterogeneity condition were indicated in a previous result compared to another treatment planning systems with C‐arm linear accelerators.^19^, ^23^ This means that the difference occurs in the way of calculating heterogeneity regions between Planning Station and Mobius3D in the PTV. The CT‐PD has only three points in Mobius3D, which are the HU:g/cc ratios: −1000:0, 0:1, and 6000:4.8. The user can adjust the CT‐PD to institutional data, but Mobius does not recommend changing the default registration. Mobius argues that “It is far more common for there to be an error in the TPS CT to density table than it is for a CT scanner to be customized to output HU values that are significantly different than other scanners.” In our institution, the CT‐PD was not observed to differ significantly.

In this phantom study, we determined that Mobius3D is more accurate than Planning Station because of two reasons: the first reason is that Mobius3D was optimized for only three treatment sites and the second reason is that Mobius3D implemented more complex parameters for MLC leakage and source size. However, to calculate dose for patient, the system needs to correctly convert to density from CT number and calculate dose in heterogeneity condition. To verify the accuracy of calculation in patients, previous study was done for Planning Station,^31^ we need further investigation using Monte Carlo simulation for Mobius3D.

There is no guideline for setting the action and tolerance levels for a 3D secondary independent verification system. Instead, it is recommended that each institution determine the proper action levels for that particular clinic using non‐IMRT MU verification.^1^ Figure 2(a), our results represent that average dose was different at each treatment site between TPS and 3D secondary independent verification. This indicates that — to set the action and tolerance levels for 3D independent verification — the user must analyze data for each treatment site and dose index. Our results are similar to previous results that analyzed differences between a TPS and Mobius3D for C‐arm linear accelerators for IMRT and non‐IMRT.^21^ All tolerance levels were within 5% total uncertainty, which is a widely accepted goal for effective radiation treatment.^32^, ^33^

Patient‐specific quality assurance needs systems to verify all treatment processes so as to accurately detect an error that could harm the patient.^34^ In addition to treatment planning process verification using Mobius3D, data transfer from TPS to linear accelerator control system and hardware malfunction during irradiation must be verified. In recent research, new approach was opposed that the system verifies the dose distribution between TPS and reconstructed dose from information generated from linear accelerator after irradiation.^24^, ^35^, ^36^, ^37^ In addition to Mobius3D, Mobius has a system using these new approaches named MobiusFX for C‐arm linear accelerators, but it does not deal with TomoTherapy now.^38^, ^39^ To secure the patient safety in TomoTherapy, we should use systems implementing these new approaches or conventional measurement‐based techniques in addition to a secondary independent dose verification system.

## 5 CONCLUSION

We described our experience with commissioning Mobius3D for TomoTherapy, and it revealed that Mobius3D has enough accuracy for the independent dose calculation system in our institution. As a result of our experience, we conclude that Mobius3D can be commissioned easily and immediately after being installed. We also indicated the action and tolerance levels for clinical implementation. A 3D secondary independent verification system gives us much more useful information about each target and OAR than we have possessed before. However, it is difficult to decide whether each plan is acceptable or not without commissioning and setting tolerances. Before clinical use, each clinic should commission the system to determine the calculation accuracy and to establish tolerances for each treatment site and dose index using treatment plans for patients who have been treated in that institution.

## CONFLICT OF INTEREST

The authors declare no conflict of interest.
